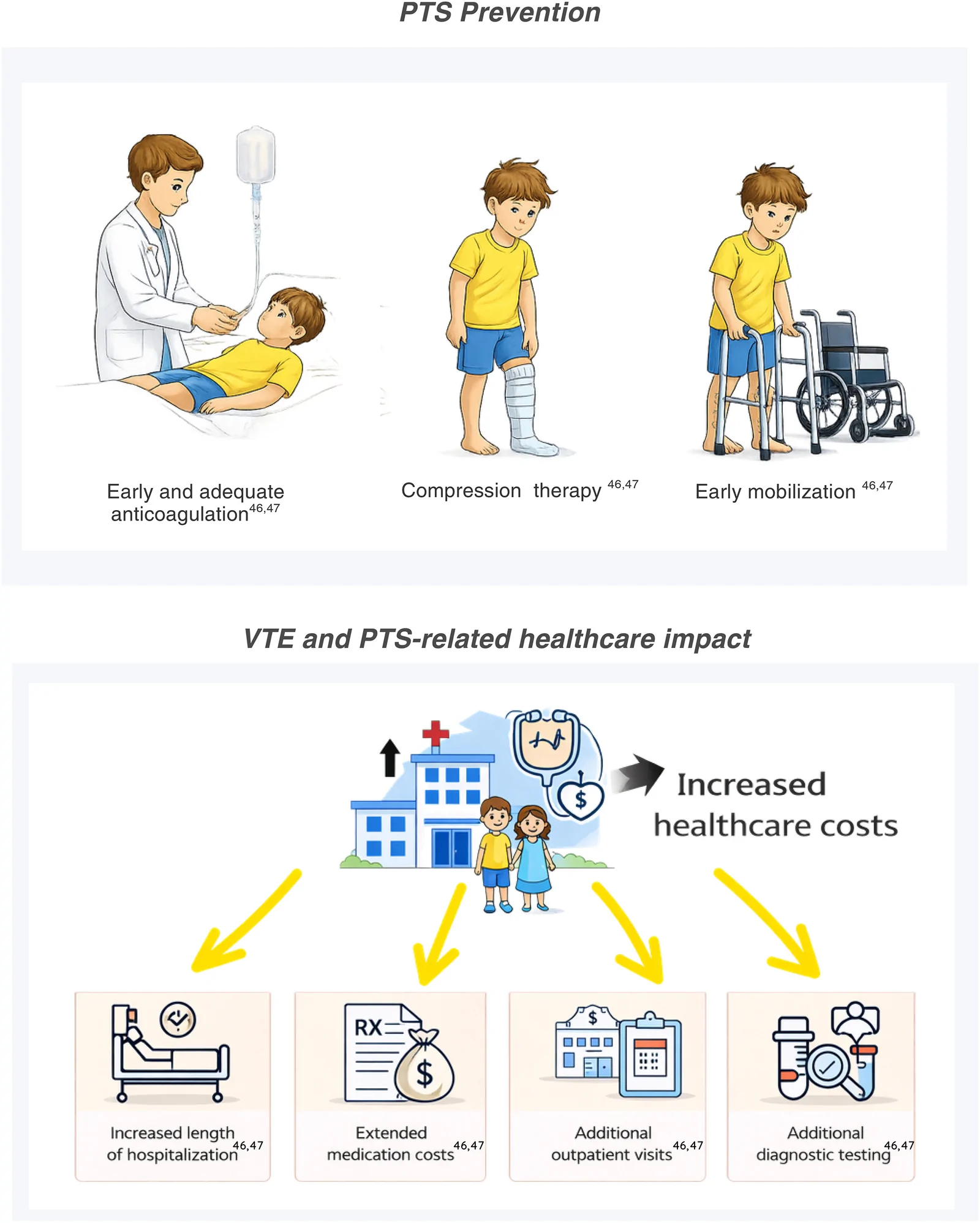# Pediatric venous thromboembolism: an illustrated review

**DOI:** 10.1016/j.rpth.2026.106892

**Published:** 2026-07-31

**Authors:** Maria G. Espanol, Maissaa Janbain

**Affiliations:** 1 Department of Pediatrics, Section of Hematology and Oncology, Tulane University School of Medicine, New Orleans, Louisiana, USA; 2 Department of Medicine, Section of Hematology and Oncology, Tulane University School of Medicine, New Orleans, Louisiana, USA; 3 Louisiana Center for Bleeding & Clotting Disorders, New Orleans, Louisiana, USA

**Keywords:** anticoagulation, deep vein thrombosis, pediatric thrombosis, pulmonary embolism, thromboprophylaxis, venous thromboemnolism

## Abstract

Venous thromboembolism (VTE) in children has historically been considered uncommon; however, it is increasingly recognized in hospitalized pediatric populations, and its incidence has risen over the past few decades. This can be attributed to several factors, including greater awareness of the condition, improved survival rates for children with chronic and complex medical conditions, increased use of supportive care (such as central venous catheters), and advancements in diagnostic imaging. Acquired conditions, such as infections, malignancies, cardiovascular diseases, and inflammatory bowel disease, are recognized as risk factors for VTE. Among these, the presence of a central venous catheter remains the most significant and common risk factor for pediatric VTE. In this review, we present and illustrate the pathophysiology and current treatment options for VTE in the pediatric population.

## Essentials

• Venous thromboembolism (VTE) incidence in children has increased in recent years.
• VTE is associated with significant morbidity and mortality.
• Early diagnosis of VTE helps prevent complications.
• Central venous catheters are a major risk factor for VTE.

## Conclusions

Significant progress has been made in the care of pediatric patients with venous thromboembolism. Current treatment approaches aim to improve outcomes, reduce long-term complications, and enhance treatment compliance.

Despite these advances, long-term complications such as postthrombotic syndrome can still occur. These complications are associated with a decreased quality of life and an increased burden on the health care system. It is essential to advocate for early diagnosis and prompt treatment of venous thromboembolism in children to help prevent these complications and improve long-term outcomes.

## Design

This illustrated review was edited using Grammarly (Grammarly Inc) for grammar, spelling, and clarity. Visuals were created using AI chatbot, BioRender.com and Canva.com.

**Figure:**
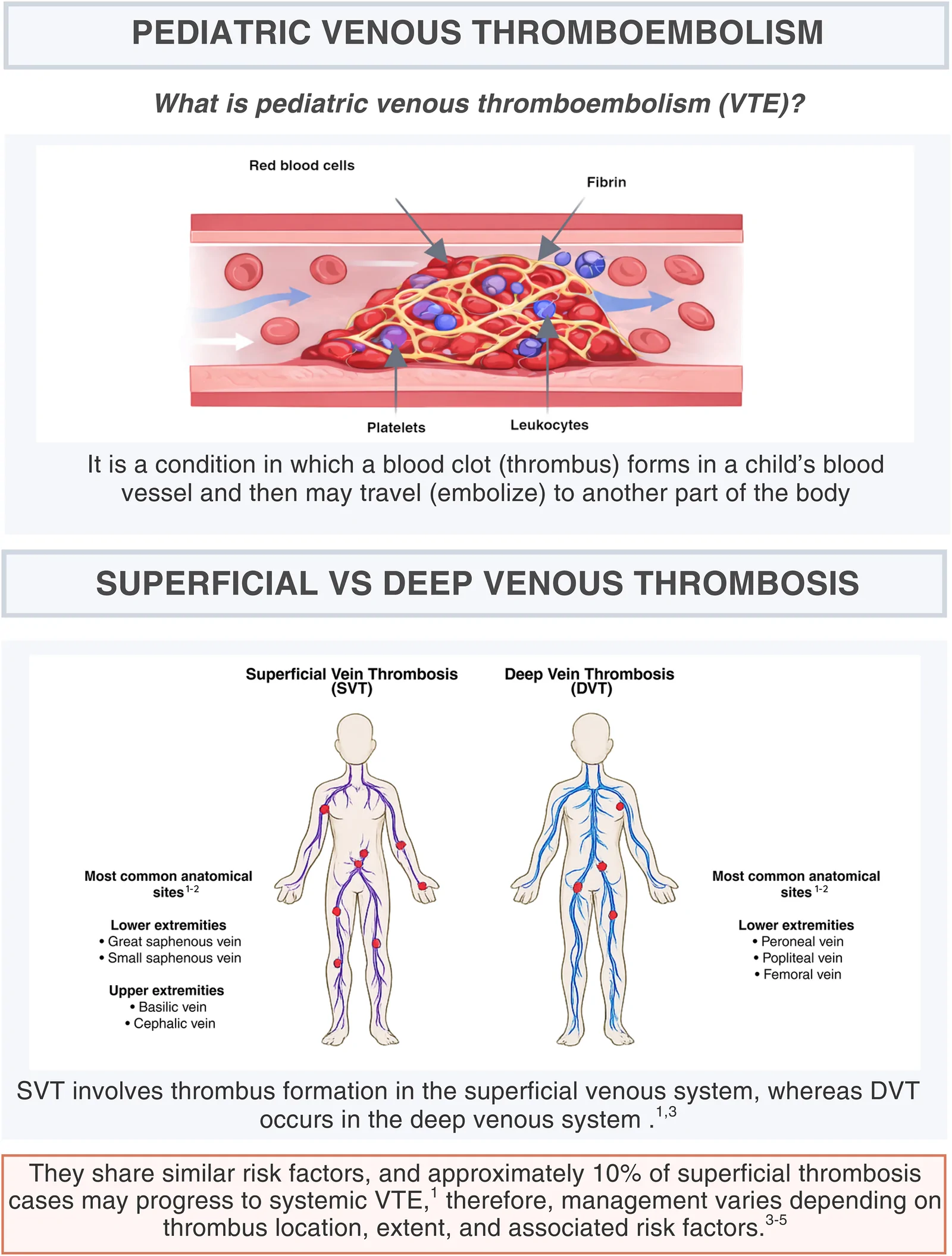

**Figure:**
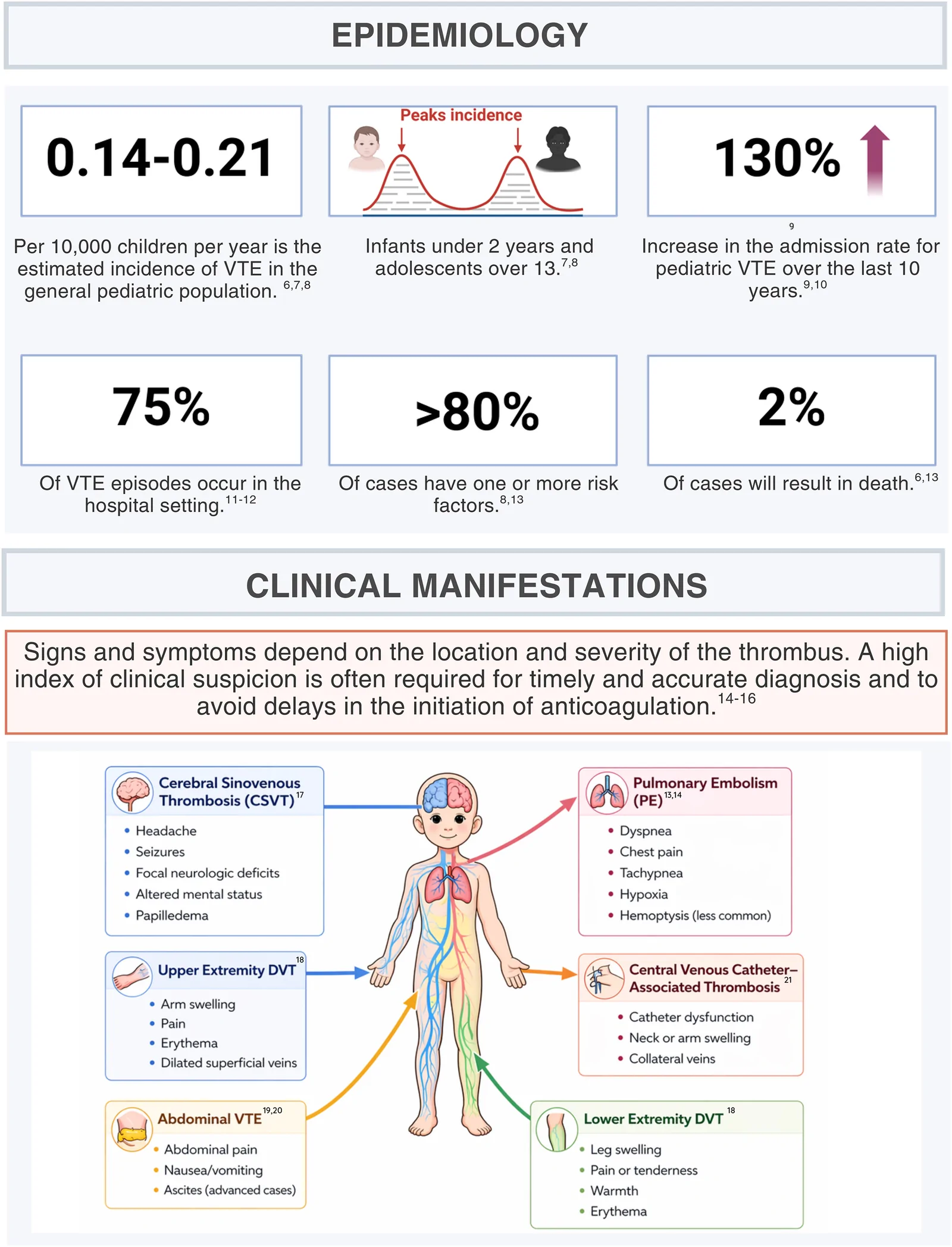

**Figure:**
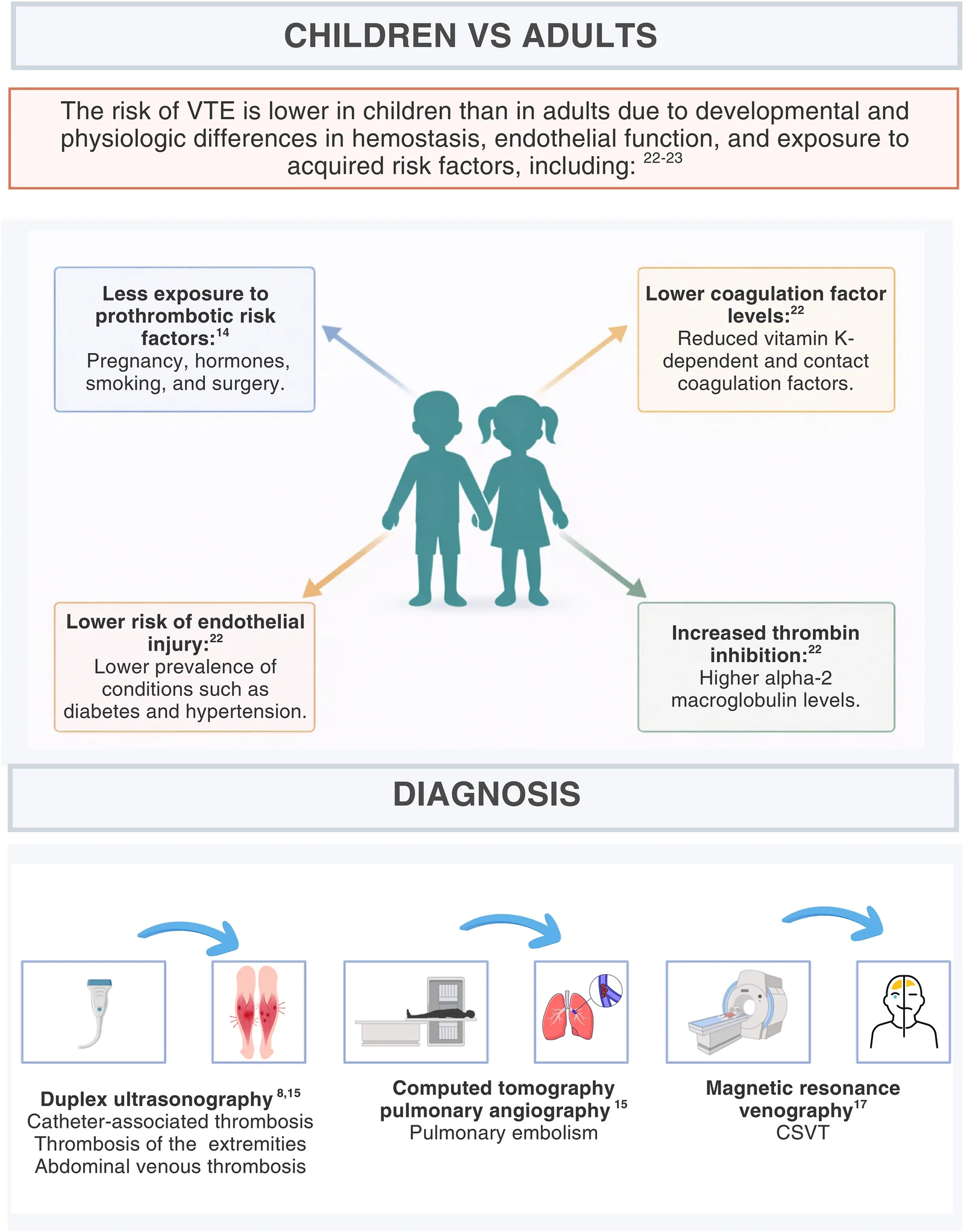

**Figure:**
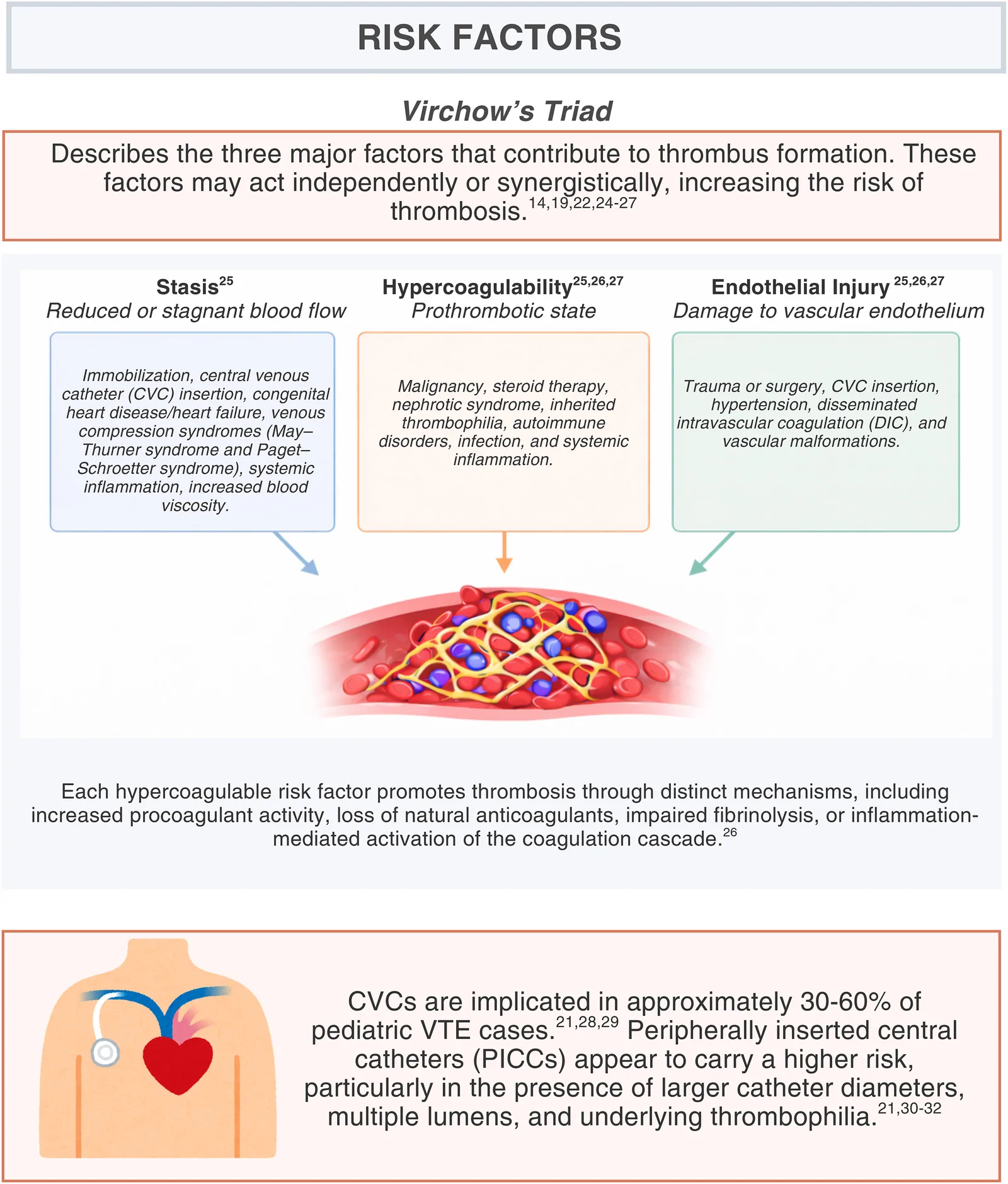

**Figure:**
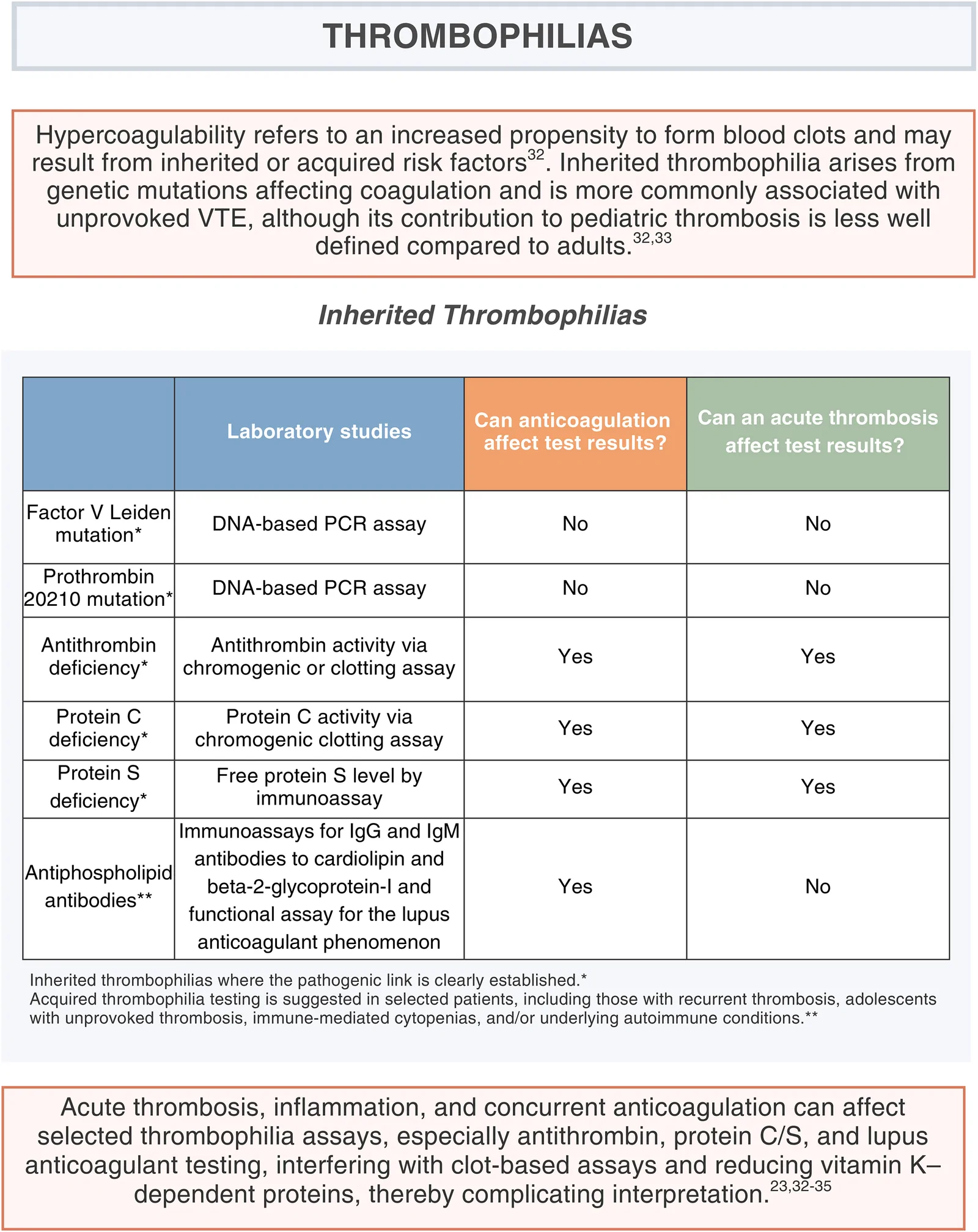

**Figure:**
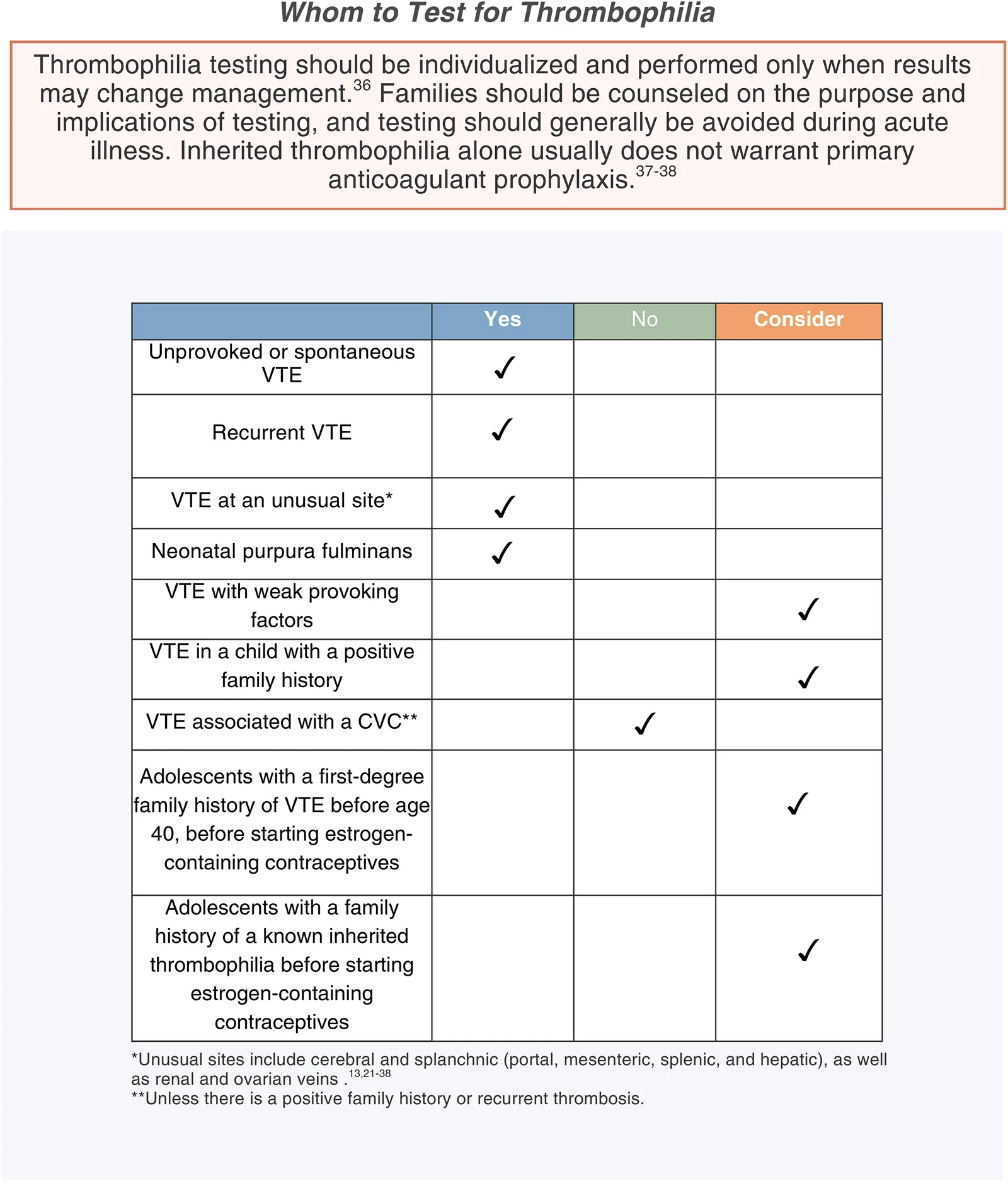

**Figure:**
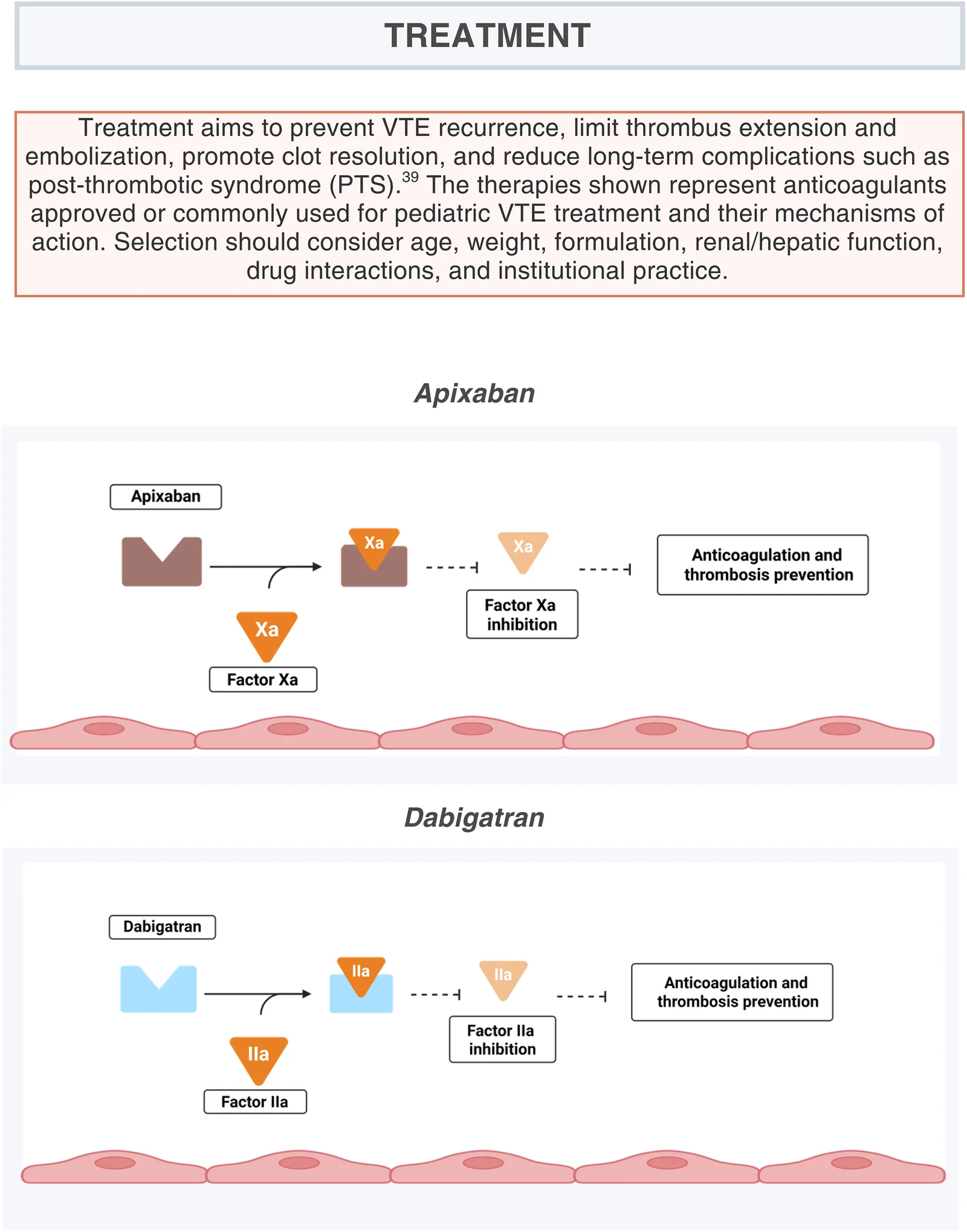

**Figure:**
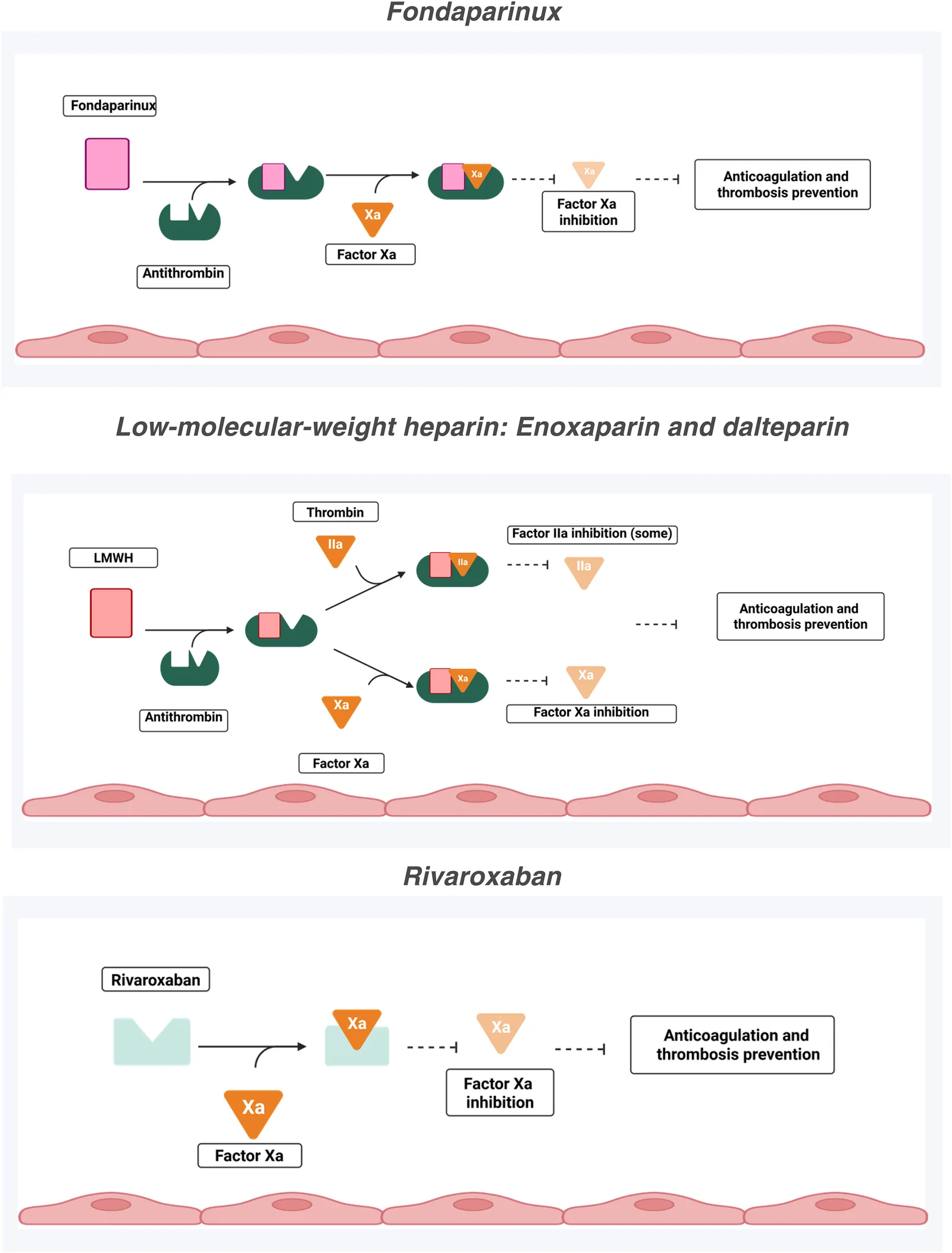

**Figure:**
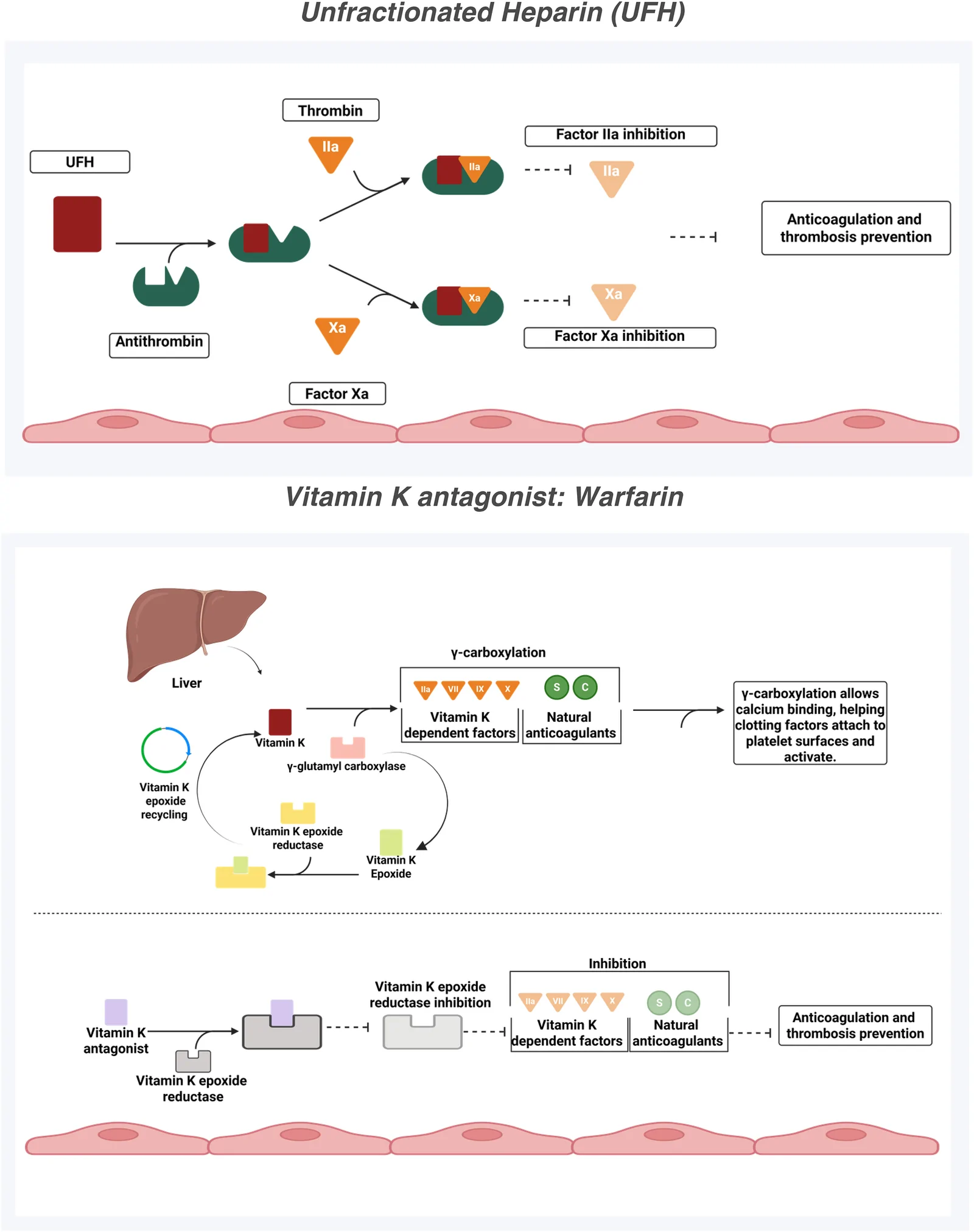

**Figure:**
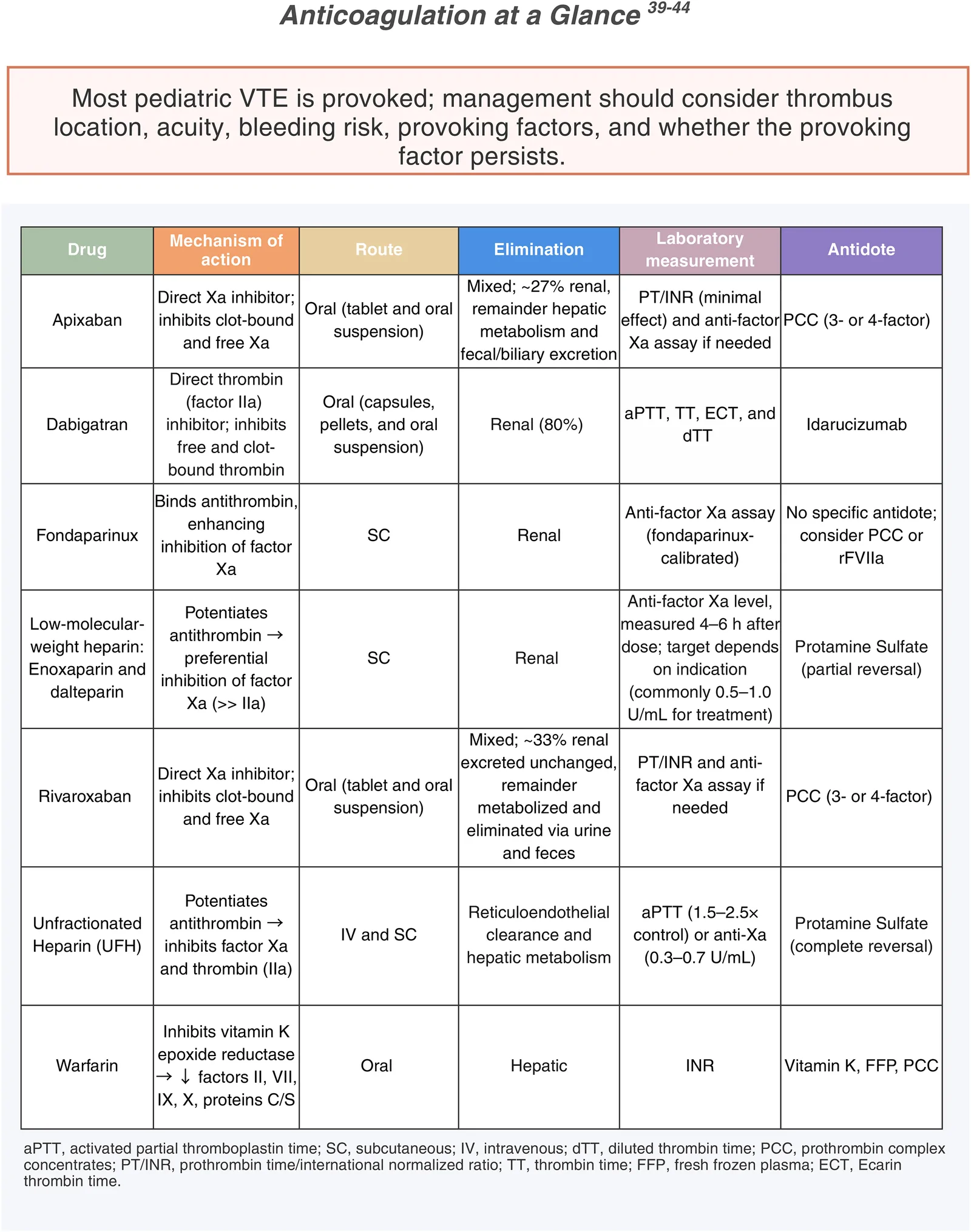

**Figure:**
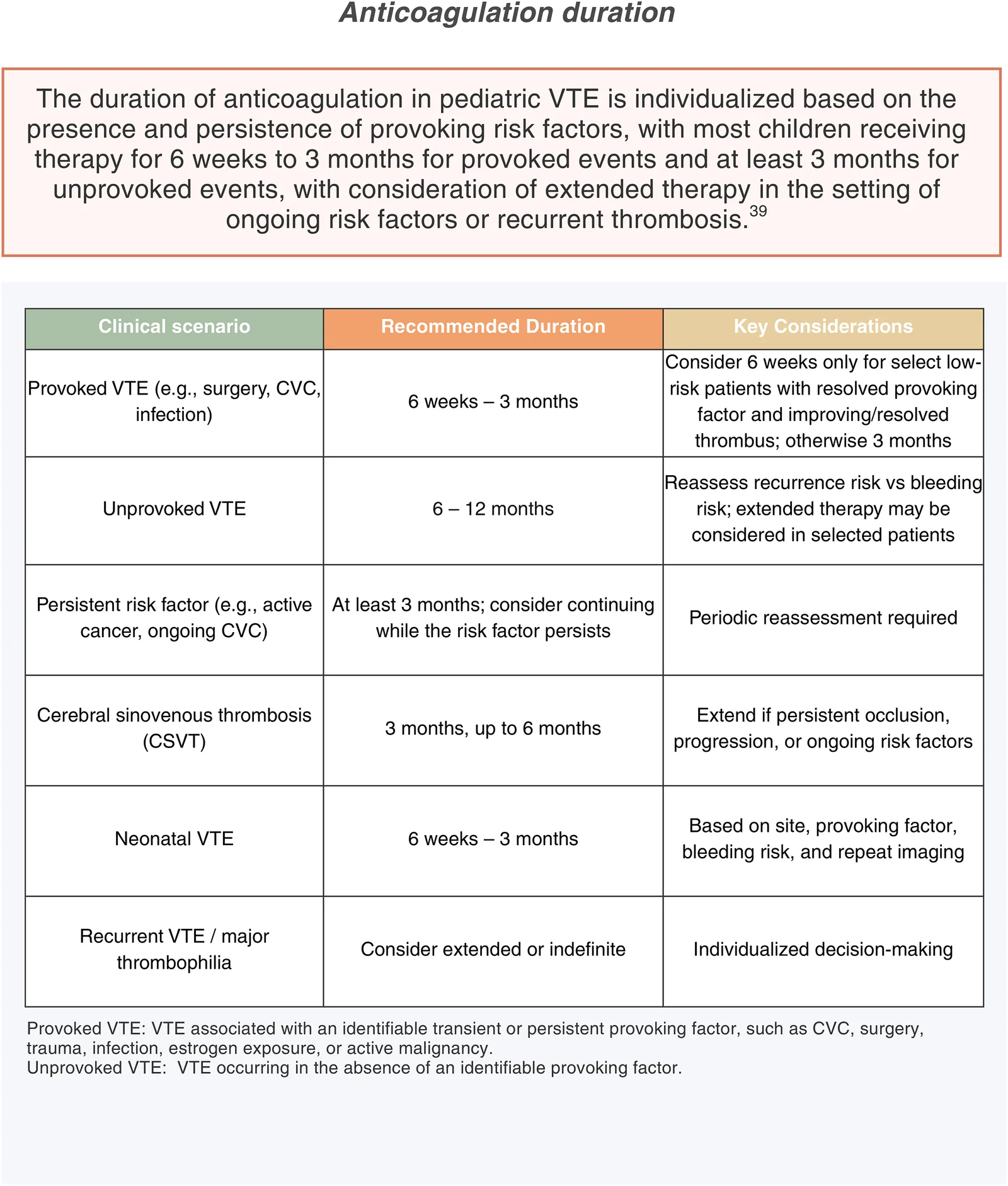

**Figure:**
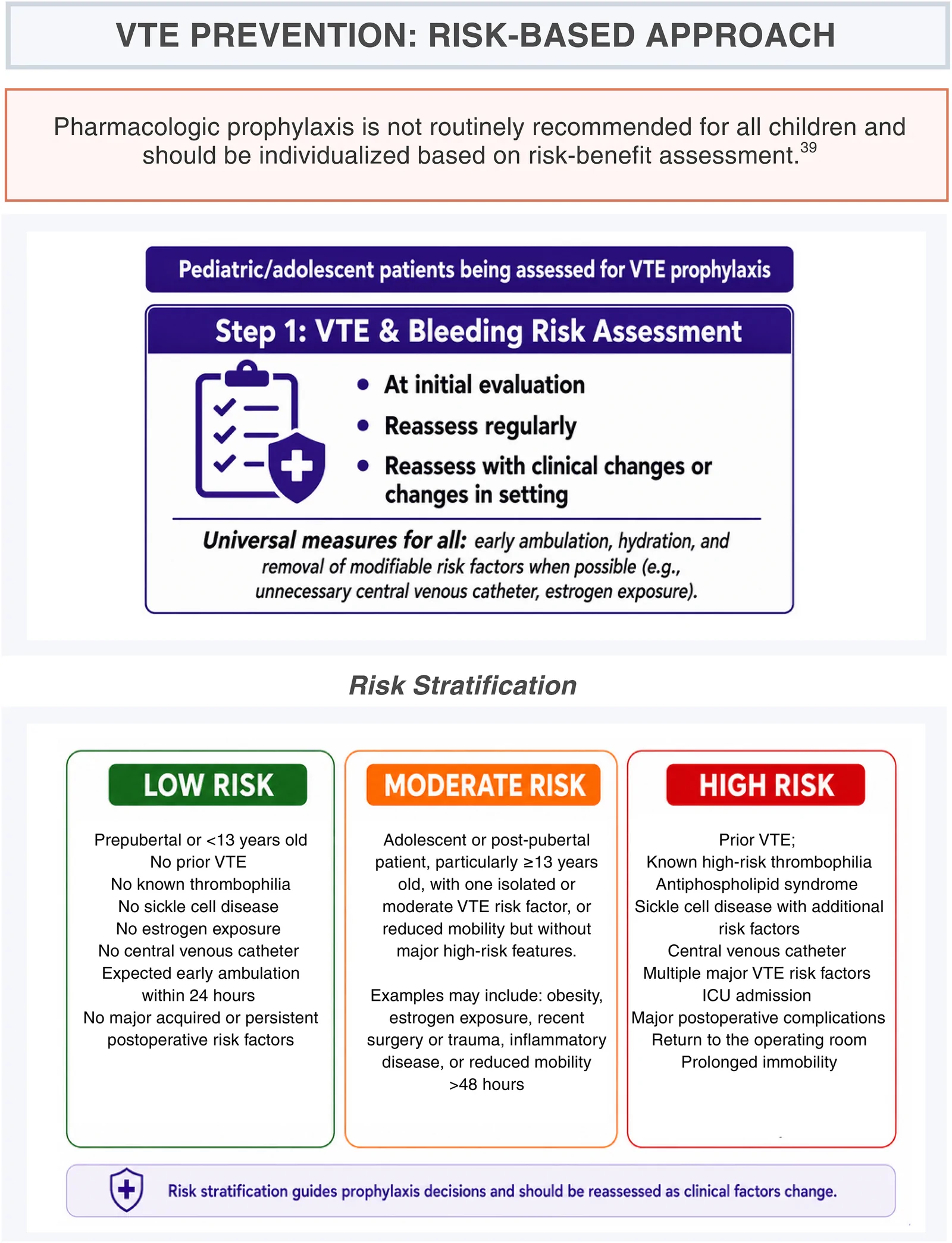

**Figure:**
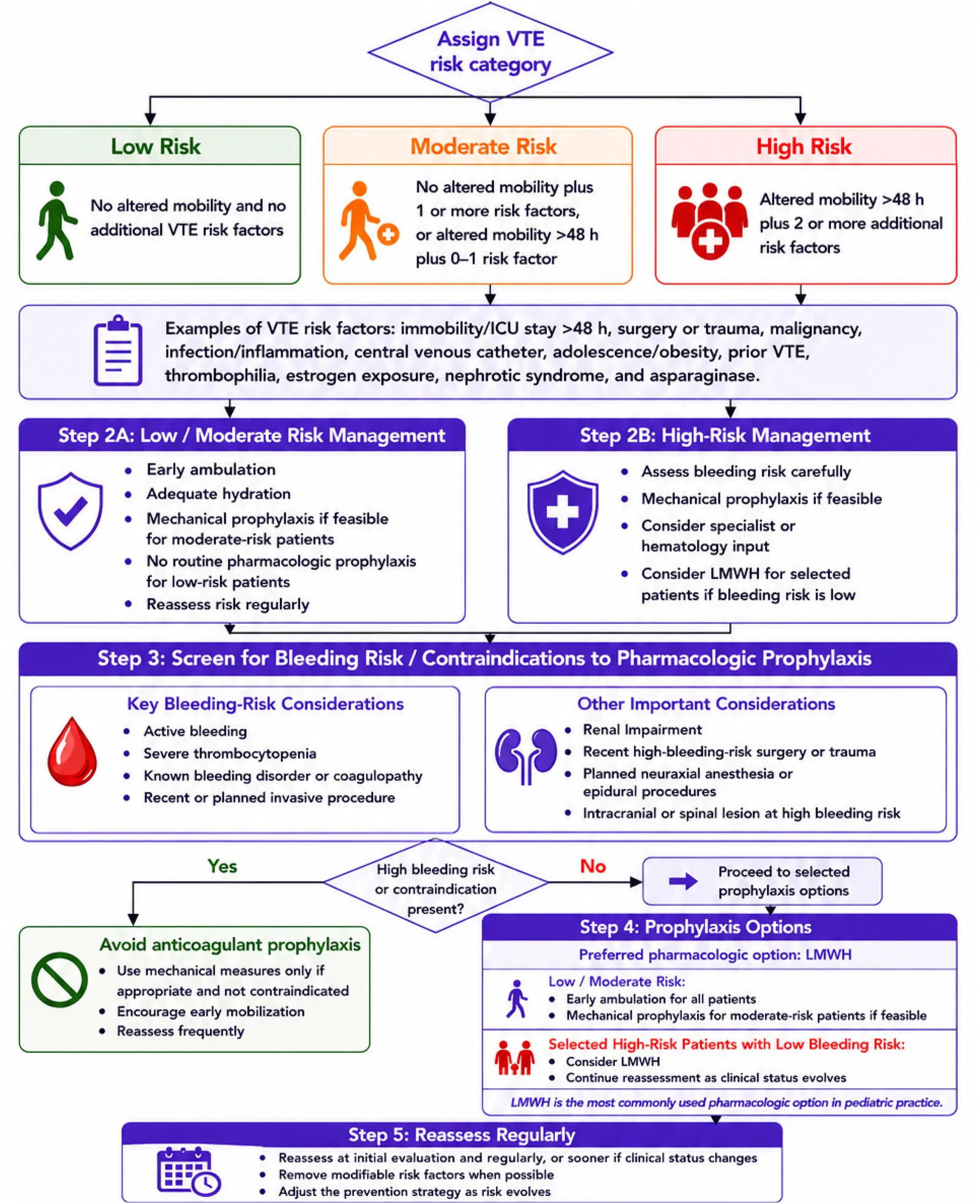

**Figure:**
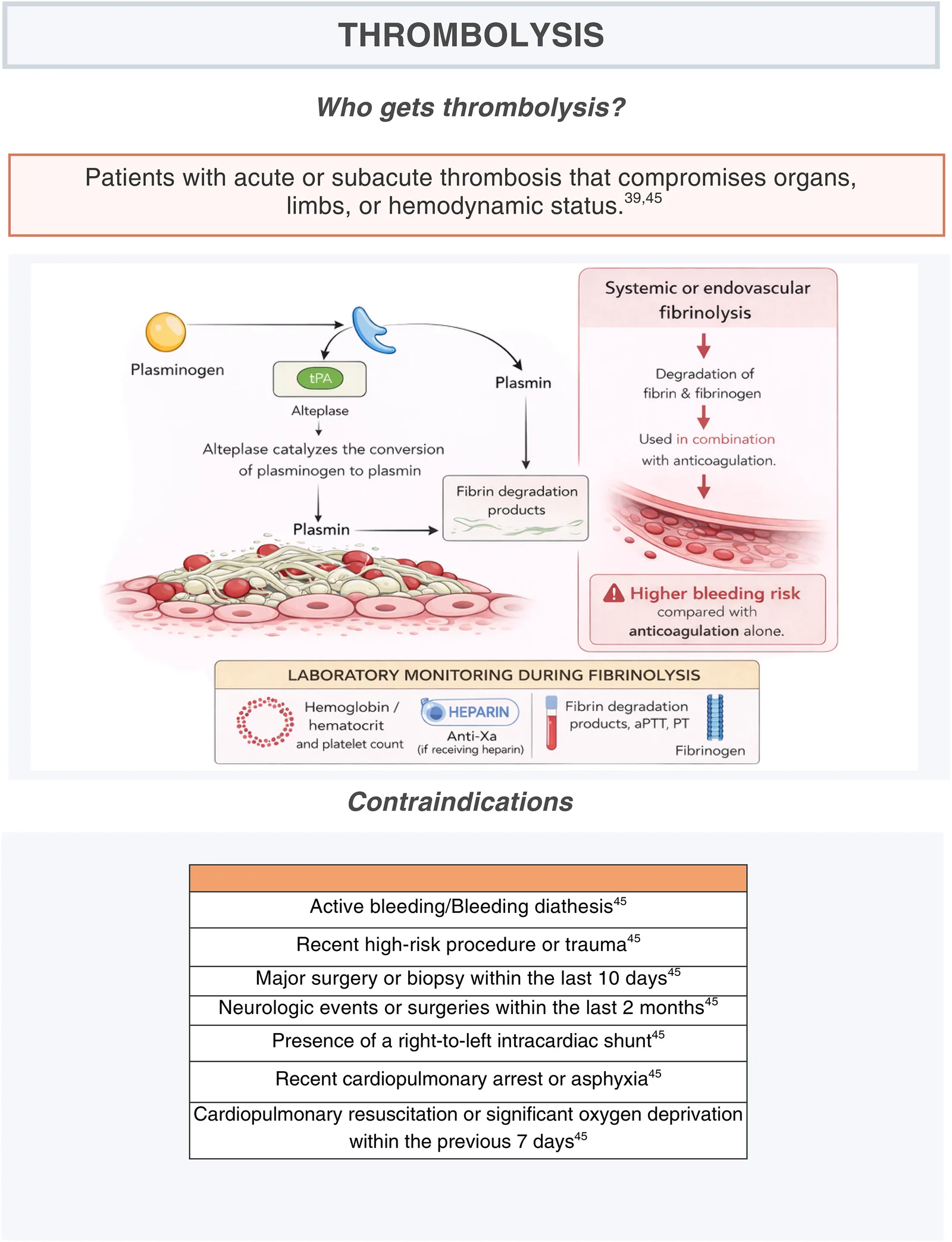

**Figure:**
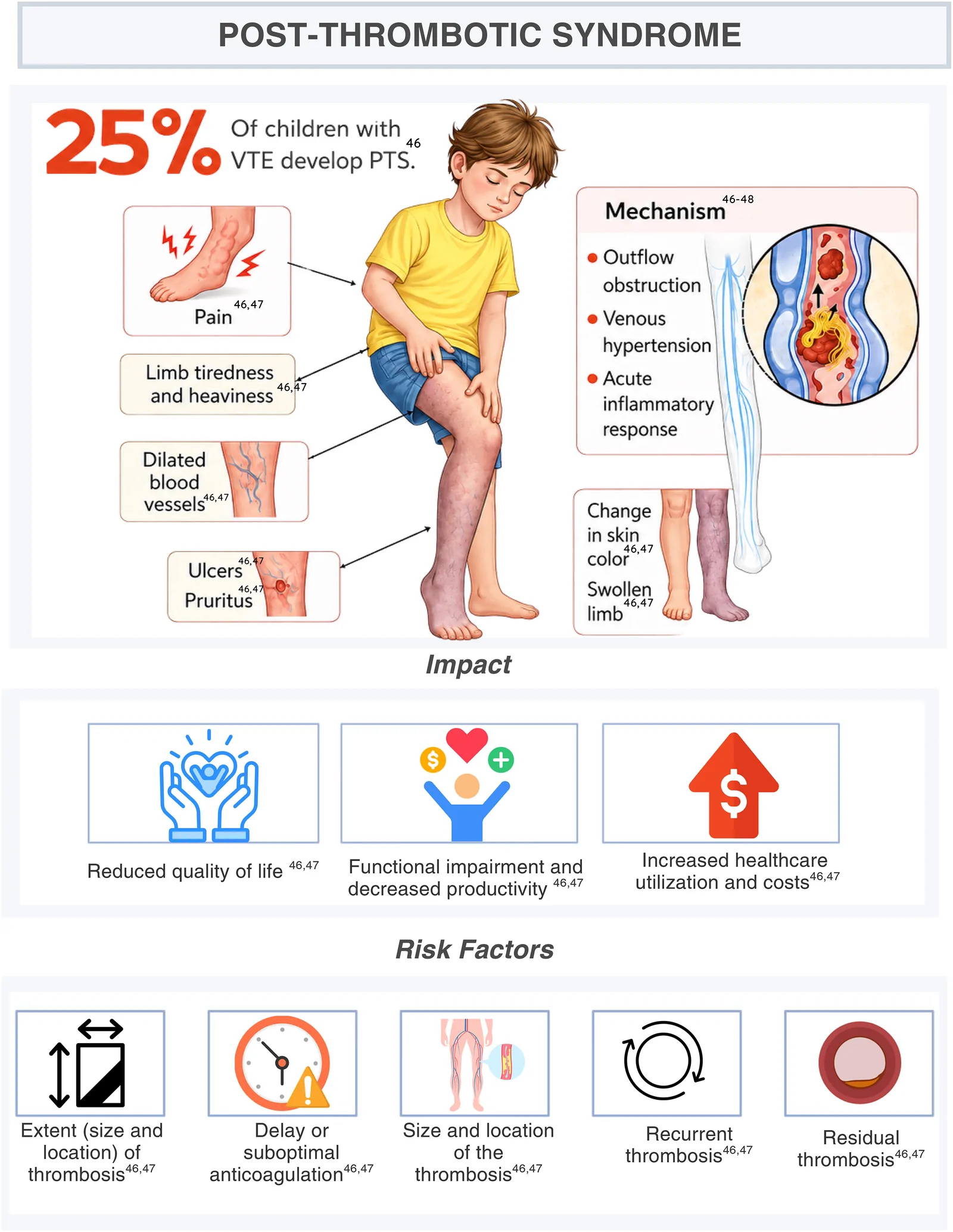

**Figure:**
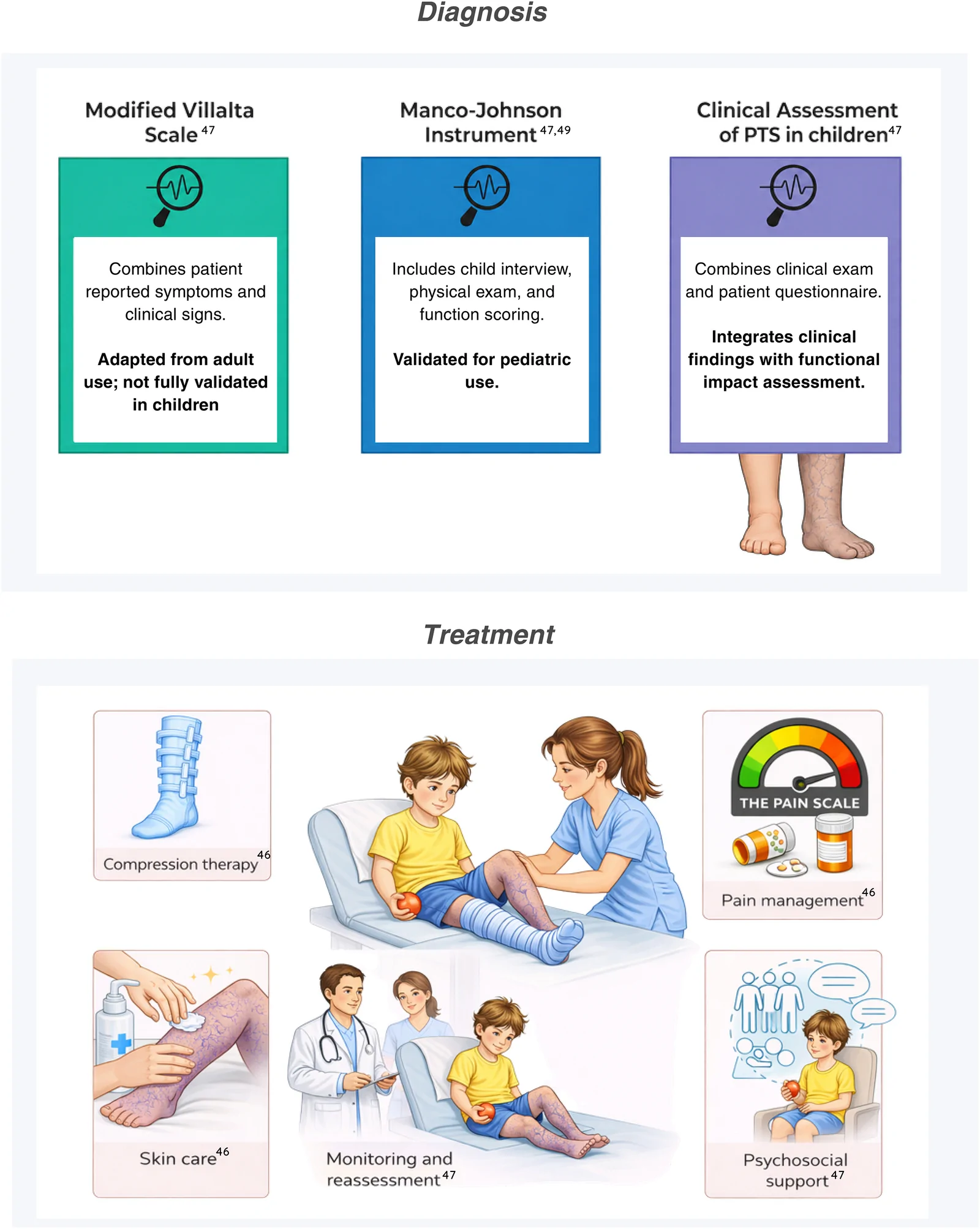

**Figure:**